# 

**Published:** 1850-07

**Authors:** 


### CONTENTS:

### PART I.—ORIGINAL COMMUNICATIONS.

Davis' Medical History, Chapter II., - - - - - - 91

ART. 1.—Brainard on Injections in Dropsical Affections, - - - I07

ART. 2.—Lescher on Milk-Sickness, - - - - - - .125

### PART II.—REVIEWS AND NOTICES OF NEW WORKS.

ART. 1.—Transactions of the American Med. Association, Instituted 1847, 130

ART. 2.—New Medical School—Progress, - · - - - 138

ART. 3.—A Universal Formulary: By R. Egglesfield Griffith, M. D., - 143

ART. 4.—Response to a Professor, and a speculation upon the Sensorium, By Bennett Dowler, M. D., - - - - - - - 141

ART. 5.—Transactions of the Medical Society of the State of New York, 141

ART. 6.—A Systematic Treatise, etc., on the Principal Diseases of the Interior Valley of North America, as they appear in the Caucassian, African, and Esquimaux Varieties of its population, by Daniel Drake, M. D., 142

### PART III.—ABSTRACTS.

ART. 1.—Spirit of the Medical Press, - - - - - - 146

### PART IV.—SELECTIONS.

ART. 1.—Tubercular Exudations into the Lungs, etc., checked by Cod-Liver Oil, - - - - - - - - 154

ART. 2.—National Medical Convention for Revising the Pharmacopœia of the United States, - - - - - - - 156

ART. 3.—Radical Cure of Corns, - - - - - - 160

ART. 4.—New Adhesive Agent, - - - - - - 161

### PART V.—EDITORIAL.

ART. 1.—American Medical Association, - - - - - 162

ART. 2·—Illinois State Medical Society, - - - - - 176

ART. 3.—Surgical Anatomy, By Joseph Maclise, M. D·, - - - 178

ART. 5.—Medical Society of Logan, Mason, and Menard Counties, Ills., 171

ART. 6.—Miscellaneous Medical Intelligence, - - - - 180

ART. 7.—Obituary, - - - - - - - - 180

